# Obesity Dilemma: Are There Enough Bariatric Surgeons?

**DOI:** 10.5812/ijem.7929

**Published:** 2012-09-30

**Authors:** Fereidoun Azizi

**Affiliations:** 1 Endocrine Research Center, Research Institute for Endocrine Sciences, Shahid Beheshti University of Medical Sciences, Tehran, IR Iran

**Keywords:** Obesity, Diabetes Mellituss Type2

The 21^st^ century epidemics of obesity and diabetes emphasize the need for an effective range of treatment option for management of these health care crisis (1).

Lack of long-term success with dieting and limited medical pharmacologic options denotes that the current medical treatment options of obesity and diabetes are far-reaching the goals of appropriate management (2) in last 2 decades, bariatric surgery has shown to be the most effective treatment for morbid obesity and diabetes in individuals with BMI ≥ 35 kg/m^2^ (3). Bariatric surgery is associated with substantial and durable weight loss and favorable metabolic effects far beyond those achieved by lifestyle modifications and pharmacological treatments, resulting in impressive reduction in comorbidant of obesity and diabetes, along with increase in longevity (4-7).

Coinideal increases in the prevelence of diabetes and obesity are issues of major concern, More than 60% of diabetics are obese and treatment of the combination of obesity and type 2 diabetes (diabesity) is now a public health priority (8). As much as 78% of diabetic patients have had complete resolution and 62% have remained in remission more than 2 years after bariatric operation (9). There are only 2 prospective randomized controlled trials in regard to bariatric surgery in diabetics. One showed 73% remission of diabetes in those who received surgery, compared to 13% in control group. Another randomized study showed that proportion of patients with a glycated hemoglobin level of 6% or less after 12 months of intervention was 12% following intensive medical therapy, 42% after gastric bypass and 37% following sleeve gastrectomy (10). Although improvement and resolution of diabetes is mostly related to weight loss after surgery, the incretin system may also have some important role. Plasma concentration of incretin hormones increases and insulin secretion and glucose tolerance improve after operation; equivalent weight loss by diet does not induce above changes (11).

Considering all of the above, bariatric surgery has emerged as an impressive treatment of obesity and type 2 diabetes. It has rapidly evolved over the last 30 years and current procedures are effective, safe, less invasive, and cost effective (12). The 1991 National Institutes of Health statement indicated that all obese patients with BMI > 40 kg/m^2^ and those with a BMI 35-40 kg/m^2^ with significant comorbidities interfering with their lifestyles, were candidates for surgical treatment (13). This statement has become the most generally accepted guideline for determining indications for bariatric surgery. As experience with surgical operations for obesity increased, few medical societies have broadened indications to include patients with BMI 30-34 kg/m^2^ with a comorbid condition that can be cured or improved by substantial weight loss (14).

Universal surgical treatment of obesity is not achievable with the world’s current health care and surgical resources. The estimation is that it would take 5500 surgeons doing 400 cases a year, each for 10 years, to attempt bariatric surgery for every 22 million obese Americans (15). In Iran, there are approximately 40 million, aged ≥ 20 years and the rates of BMI > 40 and 35-40 kg/m^2^ in this age group are 1.3 and 3.8%, respectively (16). Therefore, 520,000 subjects with BMI > 40 kg/m^2^ and 106400 type 2 diabetic patients with BMI between 35-40 require obesity operations. It may be estimated that 157 bariatric surgeons would need to do 400 cases a year, each for 10 years, to perform surgical treatment for 626400 obese Iranians. Another estimation for Tehran with its 12 million population, shows that 1.66% of people ≥ 20 years of age, have BMI > 40 kg/m^2^ (17) and another 0.69% have diabetes and BMI of 30-40 kg/m^2^ (18); hence a total of 2.35% of Tehranian population≥ aged 20 years have indications for bariatric surgery. Extrapolating these data to 7,800,000 Tehranians ≥ 20 years of age, indicates that 183,300 obese subjects may require bariatric surgery. It may then be estimated that 46 surgeons should perform 400 cases a year each for 10 years to provide surgical treatment for all obese Tehranians.

Therefore, data available indicate that bariatric surgery cannot provide the impact necessary for reduction in health care and economic costs worldwide, neither in the developed, nor in developing countries. The numbers of surgeons needed for all the operations indicated are not achievable, in particular for many developing countries, suffering from epidemics of diabesity.

It is the opinion of this editorial that although bariatric surgery is a good addition to management of patients with obesity and diabetes, the crisis caused by the rise in the prevalence of these conditions, must be addressed by more comprehensive and long-term, concerted policy efforts worldwide. Healthy eating, regulation of food supply, public education, healthy commuting through walking or biking and other means of lifestyle change need political commitment for infrastructural changes and incentives for effective research into novel and effective nonsurgical treatments for obesity and type 2 diabetes.
